# Performance Evaluation of a Maneuver Classification Algorithm Using Different Motion Models in a Multi-Model Framework

**DOI:** 10.3390/s22010347

**Published:** 2022-01-04

**Authors:** Máté Kolat, Olivér Törő, Tamás Bécsi

**Affiliations:** Department of Control for Transportation and Vehicle Systems, Budapest University of Technology and Economics, H-1111 Budapest, Hungary; mate.kolat@edu.bme.hu (M.K.); toro.oliver@kjk.bme.hu (O.T.)

**Keywords:** filtering, constraints, IMM, maneuver targeting, maneuver classification, motion models

## Abstract

Environment perception is one of the major challenges in the vehicle industry nowadays, as acknowledging the intentions of the surrounding traffic participants can profoundly decrease the occurrence of accidents. Consequently, this paper focuses on comparing different motion models, acknowledging their role in the performance of maneuver classification. In particular, this paper proposes utilizing the Interacting Multiple Model framework complemented with constrained Kalman filtering in this domain that enables the comparisons of the different motions models’ accuracy. The performance of the proposed method with different motion models is thoroughly evaluated in a simulation environment, including an observer and observed vehicle.

## 1. Introduction

Environment perception is vital for safe functionality as the decision-making layer relies solely on its awareness of the given traffic scenario. Based on the current global trend, the number of autonomous vehicles is expected to increase, requiring solutions for new types of problems [1,2]. At present, the level of a considerable number of available sensors, and thus the possible combination of sensor-fusions, can offer a wide scale in cost and robustness [3]. Furthermore, the spread of the different type of driver assistance systems create a demand for cost-effective testing methods in simulated environments [4]. Such methods have significant advantages like cost-effectiveness, test reproducibility, investigating unsafe situations, different weather, and daytime conditions [5]. Vehicle tracking evolved into a critical challenge in the automotive industry since it is necessary to design advanced driver assistance systems such as Adaptive Cruise Control, Emergency Braking System, Blind Spot Detection, or Collision Avoidance.

In the case of vehicle tracking, the next maneuver of the tracked object is unknown; therefore, it cannot be decided in advance which motion model should be used during the estimation, despite that the performance of the estimators relies highly on it [6]. Motion models can be classified by complexity, starting from linear models [7], which are the constant velocity (CV) and constant acceleration (CA) models. These models have limited capabilities for describing more complex motions; however, the probability density function is easier to handle. In the case of linear systems, from the data fusion point of view, Linear Kalman Filter or Kalman Filter (KF) is the most commonly used approach [8]. Constant Turn and Rate Velocity (CTRV) and Constant Turn Rate and Acceleration (CTRA) are more complex curvilinear models considering rotation, where Extended Kalman Filter (EKF) is a suitable solution that can handle nonlinearity. Generally, an appropriate choice of the motion model can significantly increase the vehicle tracking system’s performance. However, complexity does not always lead to a better performance [7]. There are many ways to deal with the uncertainty of the used motion model, including the Multiple Model (MM) filtering [9], where numerous filters can be designed and select the best prediction. In other words, it examines all the possible combinations of the predefined models at each timestep and returns with the best estimation. Interacting Multiple Model (IMM), which is a subtype of Multiple Model, is presented by Blom and Bar-Shalom [10] allows different filters to run parallel and described in Section 2.3. Wenkang et al. compared the traditional Square Root Cubature Kalman Filter (SCKF) to their IMM-SCKF algorithm regarding vehicle state estimation [11]. The IMM is originally proposed with Kalman filters [12], however particle filter realization also appeared in the literature [13,14,15,16,17,18].

The performance of the Kalman filter can be further increased by using state constraints. For example, suppose any boundaries limit the desired safe states. In that case, those limits can be inserted in the filtering process as state constraints and improve its performance with this additional information [19].

Kalman filter can handle state-space representations where both the measurement and the transition equations are linear with Gaussian noise. Vehicle tracking tasks can also be approached by Particle Filters (PF) [20], which is used for nonlinear and non-Gaussian problems [21]. However, it is a computationally much more expensive methodology than the Extended Kalman Filter, and any further increase in the number of particles significantly slows down the data process. Object tracking has extended literature featuring other techniques. Kowlaczuk et al. (2010) used Multiple Model Constrained Filtering with Kalman Filter for air traffic control [22]. Farmer et al. (2002) applied Interacting Multiple Model Kalman Filter for high-speed human motion tracking [23]. Zheng et al. (2009) presented a face detection method using Particle Filter [24], and Kim et al. (2020) introduced a vehicle position tracking method utilizing Lidar and radar measurements with the help of EKF [25]. Zhao et al. (2018) implemented a multi-object tracking algorithm using correlation filter [26]. Rakos et al. (2020) examined and compared various classification methods for lane change tracking [27]. Toro et al. designed a multiple object tracking algorithm, applying a particle filter-based Probability Hypothesis Density filter to track hidden but likely present objects due to occlusion [28].

Even though a significant number of Kalman Filter-based methods can be found that use the pre-fit residual, only a few apply the post-fit residual. Ormsby et al. presented a solution, where they combined the traditional pre-fit and the post-fit residual, which serves as a solution for other Multiple Model methods, like Multiple Model Adaptive Estimator (MMAE) [29]. Henderson et al. considered the carrier-phase integer ambiguity problem in the context of GPS positioning [30]. The authors used a multiple-model approach with different ambiguity hypotheses. For model likelihood computation, the post-fit residual and its covariance were used. Zhang et al. used the post-fit residual to estimate the unknown measurement noise covariance matrix [31]. The utilization of the post-fit residual increases the estimation performance and provides a better prediction for maneuver classification. Moreover, selecting the best motion model is essential, likewise considering a particular maneuver.

### Contributions of the Paper

This work examines and compares four motion models (CV, CA, CTRV, and CTRA), considering a maneuver classification method using constrained Kalman filtering with multi-model estimation. The measurements come from radar and camera sources, and the maneuvers of a road vehicle are detected and classified using various complexity of motion models. Later, the models are evaluated and compared based on their performance. The constrained filters are arranged in the structure of the interacting multiple model estimator. Constraints customize each filter to match a specific type of maneuver. The quality of a filter is determined by examining the post-fit residual.

The current study only uses linear constraints, which enables the utilization of the Kalman method. Therefore, the particle filtering approach is not necessary. On the other hand, the primary purpose, to compare the motion models, gives the same problem for all filtering paradigms.

The paper is structured as follows. Section 2 summarizes the theoretical background of the presented method. The simulation framework for testing and the maneuver detection methods are presented in Section 3. Section 4 examines the performance of the estimator. Concluding remarks are given in Section 5.

## 2. Methodology

In this study, multiple motion models are used for performance comparison purposes. Therefore, the considered system is described in double measure concerning the motion model; thus, the system can be described by a linear discrete-time dynamic model or a nonlinear model [19]. The described model using linear state transition with time index *k*:

(1) $$x_{k}=F_{k}x_{k-1}+w_{k}$$

(2) $$z_{k}=H_{k}x_{k}+v_{k}$$

where $x_{k}\in R^{n_{x}}$ is the state vector, derived from the state transitions matrix $F_{k}\in R^{n_{x}\times n_{x}}$ and $w_{k}\in R^{n_{w}}$ process noise. $z_{k}\in R^{n_{z}}$ is achieved through the observation model $Hx_{k}\in R^{n_{z}\times n_{x}}$ and observation noise $v_{k}\in R^{n_{z}}$. Both $w_{k}$ and $v_{k}$ is assumed to be a zero-mean Gaussian noise, with covariance $Q_{k}$: $w_{k}∼N\left(0,Q_{k}\right)$ and $R_{k}$: $v_{k}∼N\left(0,R_{k}\right)$. The described model uses nonlinear state transition with time index *k*:

(3) $$\begin{array}{cc} x_{k} & =f\left(x_{k-1}\right)+w_{k} \end{array}$$

(4) $$\begin{array}{cc} z_{k} & =h\left(x_{k}\right)+v_{k} \end{array}$$

where the new predicted state $x_{k+1}$ is generated from function *f* and the measurement prediction $z_{k}$ is calculated by function *h*.

### 2.1. Kalman Filter

Kalman Filter, which is the base of this study, is a linear recursive estimator [32]. For nonlinear filtering problems, the Extended Kalman Filter is an effective solution, applying the linearization through the computation of the Jacobian as the partial derivatives of the matrices. The partial derivatives of $F_{k}$ and $H_{k}$:

(5) $$F_{k}=\frac{\partial f}{\partial x}{|}_{{\hat{x}}_{k-1|k-1}}$$

(6) $$H_{k}=\frac{\partial h}{\partial x}{|}_{{\hat{x}}_{k-1|k-1}}$$

The essential concept of the Kalman Filter is the following:

(7) $$\begin{array}{cc} {\hat{x}}_{k|k-1} & =F_{k}{\hat{x}}_{k-1|k-1} \end{array}$$

(8) $$\begin{array}{cc} P_{k|k-1} & =F_{k}P_{k-1|k-1}F^{T}+G_{k}Q_{k}G_{k}^{T} \end{array}$$

(9) $$\begin{array}{cc} r_{k|k-1} & =z_{k}-H_{k}{\hat{x}}_{k|k-1} \end{array}$$

(10) $$\begin{array}{cc} S_{k|k-1} & =H_{k}P_{k|k-1}H_{k}^{T}+R_{k} \end{array}$$

(11) $$\begin{array}{cc} K_{k} & =P_{k|k-1}H_{k}^{T}S_{k|k-1}^{-1} \end{array}$$

(12) $$\begin{array}{cc} {\hat{x}}_{k|k} & ={\hat{x}}_{k|k-1}+K_{k}r_{k|k-1} \end{array}$$

(13) $$\begin{array}{cc} P_{k|k} & =\left(I-K_{k}H_{k}\right)P_{k|k-1} \end{array}$$

In (7) and (8), the a priori state estimate and the corresponding state covariance is computed, while (9) and (10) define the pre-fit residual and its covariance, where $R_{k}$ denotes the measurement noise. In (11), the Kalman gain is calculated. The *a posteriori* state estimate and error covariance are given by (12) and (13) For the Extended Kalman Filter (7) and (9) should be replaced with

(14) $$\begin{array}{cc} {\hat{x}}_{k|k-1} & =f({\hat{x}}_{k-1|k-1}) \end{array}$$

(15) $$\begin{array}{cc} r_{k|k-1} & =z_{k}-h\left({\hat{x}}_{k|k-1}\right) \end{array}$$

The estimation quality is defined by a zero-mean PDF, using the pre-fit residual and its covariance:

(16) Λ=N(r,0,S)

The estimation quality can also be defined with the help of the post-fit residual and its covariance:

(17) $$r_{k|k}=z_{k}-H_{k}{\hat{x}}_{k|k}$$

(18) $$S_{k|k}=\left(I-H_{k}K_{k}\right)S_{k|k-1}{\left(I-H_{k}K_{k}\right)}^{T}$$

### 2.2. Constrained Filtering

The Kalman filtering method is one of the most acknowledged filtering approaches; however, occasionally, it is not robust enough [33]. In different cases, like maneuver classification, filter categorization can be a necessary solution that is reachable using constraints, where the system is conditional to it, of which estimation can violate those, in case of the inadequacy of integration of constraints in the system model or the filtering process. There are numerous approaches to integrating the system model’s constraints or filtering methods, such as measurement augmentation or estimation projection. Constrained filtering can utilize this additional information and produce classes of outputs. The classification can be achieved using defined upper or lower bounds. Constraints are differentiated in several ways: equality or non-equality constraints, linear or nonlinear, soft or hard.

Linear equality constraints are formulated as follows:

(19) $$D_{k}x_{k}=d_{k}$$

and non-equality constraints:

(20) $$D_{k}x_{k}\leq d_{k}$$

where $d_{k}\in R^{n_{c}}$ is the constraint vector and $D_{k}\in R^{n_{c}\times n_{x}}$ is the constraint matrix.

#### 2.2.1. Estimation Projection

One of the solutions to the problem is the estimation projection [19]. It investigates even the state estimate fulfill a defined constraint. In this case, the state is projected in the constrained space, which is formulated as follows:

(21) $${\hat{x}}_{k|k}^{d}=\underset{x}{\mathrm{arg min}}{\left(x-{\hat{x}}_{k|k}\right)}^{⊤}W\left(x-{\hat{x}}_{k|k}\right)$$

where ${\hat{x}}_{k|k}^{d}$ is the constrained state estimate, ${\hat{x}}_{k|k}$ is the unconstrained updated estimate, and *W* is a positive definite symmetric weighting matrix, which is chosen as $P_{k|k}^{-1}$; thus, the form of the estimation is

(22) $${\hat{x}}_{k|k}^{d}={\hat{x}}_{k|k}+K_{k}^{p}\left(d_{k}-D_{k}{\hat{x}}_{k|k}\right)$$

where $K_{k}^{p}$ is

(23) $$K_{k}^{p}=P_{k|k}D_{k}^{T}{\left(D_{k}P_{k|k}D_{k}^{T}\right)}^{-1}$$

and the covariance of the constrained state estimate is

(24) $$P_{k|k}^{d}=P_{k|k}-K_{k}^{d}D_{k}P_{k|k}$$

Estimation projection can handle non-equality constraints filtering problems using the form of (20). The rows of *D* and *d* corresponding to the active constraints are chosen if *x* violates those; thus, a newly formulated constraint matrix and vector are created, with which (21) can be solved:

(25) $$D_{k}{\hat{x}}_{k|k}=d_{k}$$

#### 2.2.2. Measurement Augmentation

The other used solution for equality constraint state estimation is measurement augmentation [34]. Equality constraint (19) can be extended by additional noise ($\delta_{k}$), considering the constraints as soft constraints; thus, it has to be satisfied relatively:

(26) $$D_{k}x_{k}=d_{k}+\delta_{k}$$

The augmented measurement equation is

(27) $$\left[\begin{array}{c} z_{k}\\ d_{k} \end{array}\right]=\underset{H_{k}^{d}}{\underset{︸}{\left[\begin{array}{c} H_{k}\\ D_{k} \end{array}\right]}}x_{k}+\left[\begin{array}{c} v_{k}\\ \delta_{k} \end{array}\right]$$

or in shorter form:

(28) $$z_{k}^{d}=H_{k}^{d}x_{k}+v_{k}^{d}\;.$$

The covariance of the augmented noise term $v_{k}^{d}$ is $R_{k}^{d}$. When the noise term $\delta_{k}$ is zero, it is called Perfect Measurements (PM) and handled as hard constraints. The equations are the same as the Kalman Filter equations, but with augmented elements:

(29) $$\begin{array}{cc} r_{k|k-1}^{d} & =z_{k}^{d}-H_{k}^{d}{\hat{x}}_{k|k-1} \end{array}$$

(30) $$\begin{array}{cc} S_{k}^{d} & =H_{k}^{d}P_{k|k-1}{\left(H_{k}^{d}\right)}^{T}+R_{k}^{d} \end{array}$$

(31) $$\begin{array}{cc} K_{k}^{d} & =P_{k|k-1}{\left(H_{k}^{d}\right)}^{T}+R_{k}^{d} \end{array}$$

(32) $$\begin{array}{cc} {\hat{x}}_{k|k} & ={\hat{x}}_{k|k-1}+K_{k}^{d}r_{k|k-1}^{d} \end{array}$$

(33) $$\begin{array}{cc} P_{k|k} & =\left(I-K_{k}^{d}H_{k}^{d}\right)P_{k|k-1} \end{array}$$

### 2.3. Multi-Model Estimation

The multiple model (MM) approach helps to reduce the uncertainties of the model, which assumes that the system behaves according to one of a finite number of models [9]. The fundamental concept of the multiple model approach is to run the designed models parallel and select one with the highest performance [35]. In this study, the significant uncertainties are regarding motion models and sensors considering maneuver tracking problems.

Each model calculates its likelihood based on its constrained post-fit residual and the corresponding covariance designed using (22)–(24) in case of estimation projection as follows:

(34) $$r_{k|k}^{d}=z_{k}-H_{k}{\hat{x}}_{k|k}^{d}$$

where $r_{k|k}^{d}$ is the constrained post-fit residual. The corresponding covariance is formulated as follows:

(35) $$S_{k|k}^{d}=S_{k|k}+H_{k}K_{k}^{d}D_{k}P_{k|k}^{-1}H_{k}^{T}$$

The zero-mean PDF is defined with the help of (16). In the case of measurement augmented constraints, (29) and (30) are used for probability estimation.

### 2.4. Motion Models

**Figure 1:**
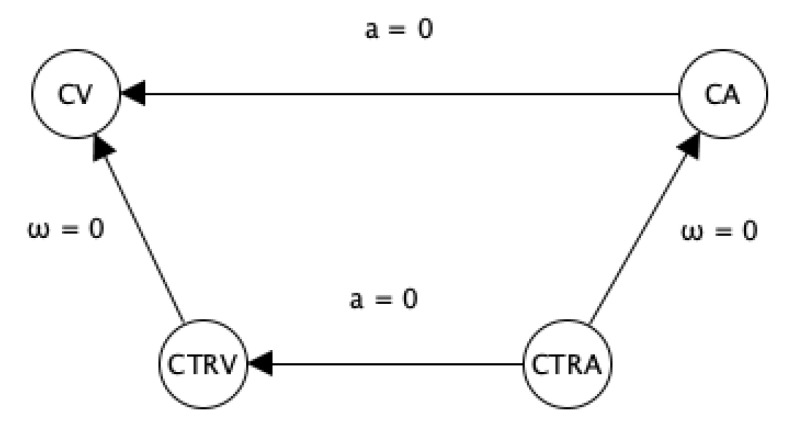
The applied motion models and their relations.

Vehicle tracking must provide a decent position estimation. The two assets of the motion models predict the vehicle’s future position and describe the dynamic behavior [7]. The performance of the estimator relies on the type of the motion model; thus, it is also feasible to compare motion models for certain applications [6]. However, it is challenging to select an appropriate motion model in advance. Motion models can be classified based on their complexity. In this study, four distinctive motion models are applied: *Constant Velocity* (CV) and *Constant Acceleration* (CA) models, which are linear models, and *Constant Turn Rate and Velocity* (CTRV) and *Constant Turn Rate and Acceleration* (CTRA), which are curvilinear models (see Figure 1).

The lowest level motion models based on their complexities are *Constant Velocity* (CV) and *Constant Acceleration* (CA) models. Linear models have the benefit of ensuring a great state probability distribution, yet both of these models presume straight motions only; thus, the state vectors are as follows:

(36) $$x_{cv}={\left(x,y,\theta ,v,\omega \right)}^{T}$$

(37) $$x_{ca}={\left(x,y,\theta ,a,v,\omega \right)}^{T}$$

where the acceleration *a* can be derived from the velocity *v*, of which lateral and longitudinal components are calculated using the heading angle θ. Yaw rate ω is ignored in linear models. Curvilinear motion models *Constant Turn Rate and Velocity* (CTRV) and *Constant Turn Rate and Acceleration* (CTRA) are on a level above, which can take into account the rotations. The state vectors are as follows:

(38) $$x_{ctrv}={\left(x,y,\theta ,v,\omega \right)}^{T}$$

(39) $$x_{ctra}={\left(x,y,\theta ,a,v,\omega \right)}^{T}$$

However, for the utilization of these models, due to their nonlinearity, a filter is needed, which can manage it. Therefore, in this study, the previously mentioned *Extended Kalman Filter* (EKF) is used for that objective.

## 3. Evaluation

**Figure 2:**
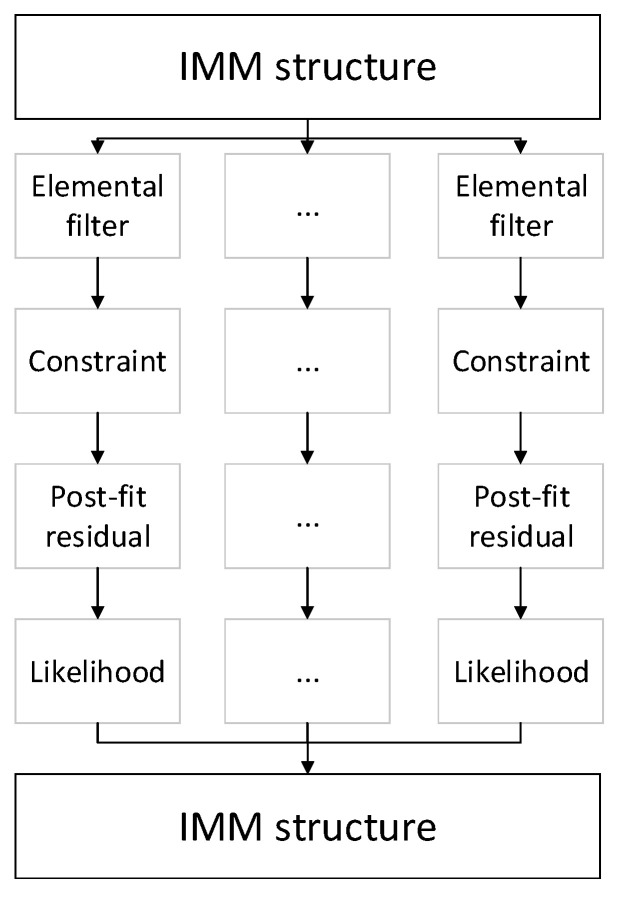
Dataflow of the proposed method.

This section presents the case study of the maneuver classification method using constrained Kalman filtering and IMM. Figure 2 shows the flowchart of the constrained IMM filter. The built scenario, the actors, the established trajectory, and the simulation environment is described in Section 3.1. The constraints corresponding to the maneuvers are introduced in Section 3.2 and the type of the constraints likewise. The estimation method and the used measurements are conferred in this subsection additionally.

### 3.1. Environment

**Figure 3:**
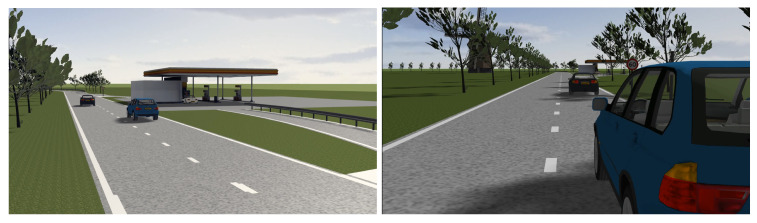
Observer (**rear**) and maneuvering vehicle (**front**) in the simulation.

The study is implemented in Simulink, using Automated Driving Toolbox provided by Matlab. In the scenario, two actors take place (see Figure 3). The observed vehicle moves along a predefined trajectory performing various maneuvers. The observer moves behind and predicts the maneuvers using radar and camera measurements. Two types of maneuvers can be distinguished corresponding to the lateral motion and the longitudinal, are described in Table 1 and Table 2.

The observer vehicle moves behind and takes measures via radar. Moreover, the observer vehicle also detects the lane line using camera information; therefore, it can describe the used lane line. The radar takes measurements in polar coordinates; thus, the collected information is the angle γ, velocity *v*, and distance *r* between the two actors. The state space is represented in various forms based on the used motion model. Four types of motion models are used and compared in this study: the previously mentioned CV, CA, CTRV, and CTRA. The state-space representations are described in (36)–(39); therefore, the radar measurements $z_{r}=\left[\gamma ,v,r\right]$ transformation is mandatory to be consistent with the state vector during the filtering process. The distance part of the measurement vector and the state-vector is not identical; therefore, the associated part of the covariance matrix $R_{1}=\mathrm{diag}\left(\sigma_{\theta }^{2},\sigma_{r}^{2}\right)$ is needed to be transformed likewise. The corresponding covariance is calculated using the Jacobian polar to Cartesian transformation:

(40) $$R^{p}=JR_{1}J^{T}$$

(41) $$J=\left[\begin{array}{cc} -r\sin\theta & \cos\theta \\ r\cos\theta & \sin\theta \end{array}\right]$$

and

(42) $$R^{p}=\left[\begin{array}{cc} \sigma_{r}^{2}\cos^{2}\theta +\sigma_{\theta }^{2}r^{2}\sin\theta & (\sigma_{r}^{2}-\sigma_{\theta }^{2}r^{2})\cos\theta \sin\theta \\ (\sigma_{r}^{2}-\sigma_{\theta }^{2}r^{2})\cos\theta \sin\theta & \sigma_{\theta }^{2}r^{2}\cos^{2}\theta +\sigma_{r}^{2}\sin\theta \end{array}\right]$$

The yaw rate is derived from camera information, which allows detecting lane line as a polynomial. Camera measures the curvature derivative $\dot{\kappa }$, curvature κ, the heading angle θ and the lateral offset $y_{lat}$; thus, $z^{c}=\left[\dot{\kappa },\kappa ,\theta ,y_{lat}\right]$. Yaw rate ω is calculated using the camera measurement of curvature derivative and curvature. Moreover, the distance measure of the radar sensor is mandatory likewise.

Yaw rate ω is calculated as follows:

(43) $$\omega =\dot{\rho }v$$

where *v* is the doppler velocity and $\dot{\rho }$ is

(44) $$\dot{\rho }=\dot{\kappa }x+\kappa$$

where *x* is the longitudinal component of the distance measurement.

For each motion model, position error and maneuver probabilities are calculated. The maneuver probabilities are collected using IMM, which allows running the corresponding filters parallel, where each filter exemplifies a constrained specified maneuver. The filters are implemented in Simulink. The state transition matrices *F* and matrices *H* are the following, where T=0.1 s is the sampling time:

(45) $$F^{CV}=\left[\begin{array}{ccccc} 1 & 0 & -Tv\sin\theta & T\cos\theta & 0\\ 0 & 1 & Tv\cos\theta & T\sin\theta & 0\\ 0 & 0 & 1 & 0 & 0\\ 0 & 0 & 0 & 1 & 0\\ 0 & 0 & 0 & 0 & 0 \end{array}\right]$$

(46) $$H^{CV}=\left[\begin{array}{ccccc} 1 & 0 & 0 & 0 & 0\\ 0 & 1 & 0 & 0 & 0\\ 0 & 0 & 0 & 1 & 0\\ 0 & 0 & 0 & 0 & 0 \end{array}\right]$$

(47) $$F^{CA}=\left[\begin{array}{cccccc} 1 & 0 & -Tv\sin\theta & \frac{T^{2}}{2}\cos\theta & T\cos\theta & 0\\ 0 & 1 & Tv\cos\theta & \frac{T^{2}}{2}\sin\theta & T\sin\theta & 0\\ 0 & 0 & 1 & 0 & 0 & 0\\ 0 & 0 & 0 & 1 & 0 & 0\\ 0 & 0 & 0 & T & 1 & 0\\ 0 & 0 & 0 & 0 & 0 & 0 \end{array}\right]$$

(48) $$H^{CA}=\left[\begin{array}{cccccc} 1 & 0 & 0 & 0 & 0 & 0\\ 0 & 1 & 0 & 0 & 0 & 0\\ 0 & 0 & 0 & 0 & 1 & 0\\ 0 & 0 & 0 & 0 & 0 & 0 \end{array}\right]$$

The yaw rate ω is ignored by the constant velocity and constant acceleration motion models; hence, the 0 value does not evolve through the state transition matrix *F* and matrix *H*. Furthermore, heading angle θ and acceleration *a* are not measured, only predicted; thus, ignored likewise in matrix *H*.

The state transition *F* and measurement *H* matrices for the CTRV model are

(49) $$F^{CTRV}=\left[\begin{array}{ccccc} 1 & 0 & \frac{\partial f_{x}^{CTRV}}{\partial \theta } & \frac{\partial f_{x}^{CTRV}}{\partial v} & \frac{\partial f_{x}^{CTRV}}{\partial \omega }\\ 0 & 1 & \frac{\partial f_{y}^{CTRV}}{\partial \theta } & \frac{\partial f_{y}^{CTRV}}{\partial v} & \frac{\partial f_{y}^{CTRV}}{\partial \omega }\\ 0 & 0 & 1 & 0 & T\\ 0 & 0 & 0 & 1 & 0\\ 0 & 0 & 0 & 0 & 1 \end{array}\right]$$

where the partial derivatives are

(50) $$\begin{array}{cc} \frac{\partial f_{x}^{CTRV}}{\partial \theta } & =\frac{v}{\omega }\cos\left(\theta +\omega T\right)-\frac{v}{\omega }\cos\theta \end{array}$$

(51) $$\begin{array}{cc} \frac{\partial f_{x}^{CTRV}}{\partial v} & =\frac{\sin(\theta +\omega T)-\sin\theta )}{\omega } \end{array}$$

(52) $$\begin{array}{cc} \frac{\partial f_{x}^{CTRV}}{\partial \omega } & =\frac{v}{\omega^{2}}\left(\cos\left(\theta +\omega T\right)+\omega T\sin\left(\theta +\omega T\right)-\cos\theta \right) \end{array}$$

(53) $$\begin{array}{cc} \frac{\partial f_{y}^{CTRV}}{\partial \theta } & =\frac{v}{\omega }\sin\left(\theta +\omega T\right)-\frac{v}{\omega }\sin\theta \end{array}$$

(54) $$\begin{array}{cc} \frac{\partial f_{y}^{CTRV}}{\partial v} & =\frac{\cos\theta -\cos(\theta +\omega T)}{\omega } \end{array}$$

(55) $$\begin{array}{cc} \frac{\partial f_{y}^{CTRV}}{\partial \omega } & =\frac{v}{\omega^{2}}\left(\cos\left(\theta +\omega T\right)+\omega T\sin\left(\theta +\omega T\right)-\cos\theta \right) \end{array}$$

and

(56) $$H^{CTRV}=\left[\begin{array}{ccccc} 1 & 0 & 0 & 0 & 0\\ 0 & 1 & 0 & 0 & 0\\ 0 & 0 & 0 & 1 & 0\\ 0 & 0 & 0 & 0 & 1 \end{array}\right]\;.$$

The state transition *F* and measurement *H* matrices for the CTRA model are

(57) $$F^{CTRA}=\left[\begin{array}{cccccc} 1 & 0 & \frac{\partial f_{x}^{CTRA}}{\partial \theta } & \frac{\partial f_{x}^{CTRA}}{\partial a} & \frac{\partial f_{x}^{CTRA}}{\partial v} & \frac{\partial f_{x}^{CTRA}}{\partial \omega }\\ 0 & 1 & \frac{\partial f_{y}^{CTRA}}{\partial \theta } & \frac{\partial f_{y}^{CTRA}}{\partial a} & \frac{\partial f_{y}^{CTRA}}{\partial v} & \frac{\partial f_{y}^{CTRA}}{\partial \omega }\\ 0 & 0 & 1 & 0 & 0 & T\\ 0 & 0 & 0 & 1 & 0 & 0\\ 0 & 0 & 0 & T & 1 & 0\\ 0 & 0 & 0 & 0 & 0 & 1 \end{array}\right]$$

where the partial derivatives are

(58) $$\begin{array}{cc} \frac{\partial f_{x}^{CTRA}}{\partial \theta } & =\frac{a(\sin\theta -\sin(\theta +\omega T))+\omega (aT+v)\cos(\theta +\omega T)-v\omega \cos\theta }{\omega^{2}} \end{array}$$

(59) $$\begin{array}{cc} \frac{\partial f_{x}^{CTRA}}{\partial a} & =\frac{-\cos\theta +T\omega \sin(\theta +\omega T)+\cos(\theta +\omega T)}{\omega^{2}} \end{array}$$

(60) $$\begin{array}{cc} \frac{\partial f_{x}^{CTRA}}{\partial v} & =\frac{\sin(\theta +\omega T)-\sin\theta }{\omega } \end{array}$$

(61) $$\begin{array}{cc} \frac{\partial f_{x}^{CTRA}}{\partial \omega } & =\frac{2a\cos\theta +(T\omega^{2}\left(aT+v\right)-2a)\cos\left(\theta +\omega T\right)-\omega \left(2aT+v\right))\sin\left(\theta +\omega T\right)+v\omega \sin\theta }{\omega^{3}} \end{array}$$

(62) $$\begin{array}{cc} \frac{\partial f_{y}^{CTRA}}{\partial \theta } & =\frac{-a(\cos\theta +a\cos(\theta +\omega T))+\omega (aT+v)\sin(\theta +\omega T)+v\sin(\theta +\omega T)-v\sin\theta }{\omega^{2}} \end{array}$$

(63) $$\begin{array}{cc} \frac{\partial f_{y}^{CTRA}}{\partial a} & =-\frac{\sin\theta +T\omega \cos(\theta +\omega T)-\sin(\theta +\omega T)}{\omega^{2}} \end{array}$$

(64) $$\begin{array}{cc} \frac{\partial f_{y}^{CTRA}}{\partial v} & =\frac{\cos\theta -\cos(\theta +\omega T)}{\omega } \end{array}$$

(65) $$\begin{array}{cc} \frac{\partial f_{y}^{CTRA}}{\partial \omega } & =\frac{2a\sin\theta +(T\omega^{2}\left(aT+v\right)-2a)\sin\left(\theta +\omega T\right)+\omega \left(2aT+v\right))\cos\left(\theta +\omega T\right)-v\omega \cos\theta }{\omega^{3}} \end{array}$$

and

(66) $$H^{CTRA}=\left[\begin{array}{cccccc} 1 & 0 & 0 & 0 & 0 & 0\\ 0 & 1 & 0 & 0 & 0 & 0\\ 0 & 0 & 0 & 0 & 1 & 0\\ 0 & 0 & 0 & 0 & 0 & 1 \end{array}\right]\;.$$

Curvilinear motion models (CTRV, CTRA) allow yaw rate and require nonlinear filters; therefore, Extended Kalman Filter is used. Thus, linearization is mandatory, which is described above. The heading angle θ and acceleration *a* is ignored likewise in the case of linear models. The noise for the motion model is a discrete white noise, which is denoted by covariance *Q*:

(67) $$Q=\Gamma q\Gamma^{T}$$

where Γ is the known disturbance matrix:

(68) $$\Gamma =\left[\begin{array}{cccc} 0 & 0 & 0 & 0\\ 0 & 0 & 0 & 0\\ 0 & 0 & T & \frac{T^{2}}{2}\\ T & 0 & 0 & 0\\ \frac{T^{2}}{2} & T & 0 & 0\\ 0 & 0 & 0 & T \end{array}\right]$$

and *T* is the sampling interval.

### 3.2. Constraints

Three lateral (Table 3) and four longitudinal (Table 4) maneuvers are introduced in this study. In the constraints of the lateral maneuvers (Table 5), upper and lower bounds are applied to the lateral motions as follows:

Four distinctive longitudinal maneuvers are introduced in the study based on the distance and velocity difference as follows:

Velocity and distance constraints are applied in various longitudinal maneuvers. Losing distance occurs when the observer is faster with at least 1 $m\;\cdot \;s^{-1}$ and the distance between the two actors is more than 10 m. Collision warning arises when the velocity difference is minimum −1 $m\;\cdot \;s^{-1}$, and the distance is maximum 10 m. Distance keeping is implemented as a measurement augmented soft constraint; thus, the estimation is not required to be explicitly zero, only approximately. Gaining distance occurs when the velocity difference is at least 1 $m\;\cdot \;s^{-1}$.

The longitudinal constraints are implemented as follows (Table 6):

## 4. Results

The initial velocity of the observed vehicle is 30 $m\;\cdot \;s^{-1}$ and speed up subsequently to 35 $m\;\cdot \;s^{-1}$. The observer vehicle has an initial velocity of 35 $m\;\cdot \;s^{-1}$, which firstly slows down to 30 $m\;\cdot \;s^{-1}$, then accelerates back to 35 $m\;\cdot \;s^{-1}$. The motion models and the corresponding filters are evaluated in two aspects. Generally, seven filters run parallel, separated into two classes. Three filters agree to the lateral maneuvers and four to the longitudinals using the same motion models, and each model is evaluated regarding maneuver classification and compared. Moreover, each motion model is assessed by position and velocity error likewise. The mode transition probabilities are designed as

(69) $$\pi_{lat}=\left[\begin{array}{ccc} 0.7 & 0.2 & 0.1\\ 0.15 & 0.15 & 0.7\\ 0.1 & 0.2 & 0.7 \end{array}\right]$$

where $\pi_{lat}$ is the mode transition matrix corresponding to the lateral maneuvers. The first row and column are associated with the progressing in the left lane, the second is the lane changing maneuver, and the last is the right lane. The mode transition matrix regarding the longitudinal maneuvers $\pi_{lon}$ is designed as follows:

(70) $$\pi_{lon}=\left[\begin{array}{cccc} 0.67 & 0.01 & 0.31 & 0.01\\ 0.01 & 0.58 & 0.11 & 0.30\\ 0.21 & 0.21 & 0.57 & 0.01\\ 0.01 & 0.21 & 0.21 & 0.57 \end{array}\right]$$

Finding the correct parameters for the transition matrix always represents a significant concern. Consequently, trial and error based heuristic searches are conducted to capture the proper parameters for our research.

**Figure 4:**
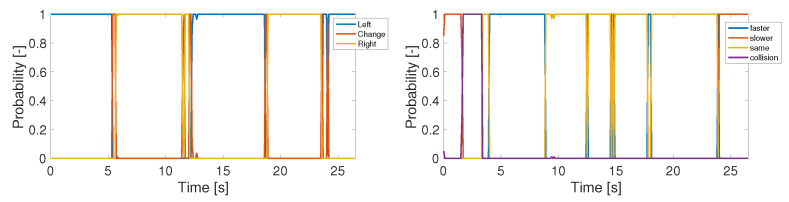
Probabilities of the investigated maneuvers using CTRA model.

Losing distance corresponding to the first row and column corresponds to a gaining distance maneuver associated with the second, following the distance keeping prerogative. The last row and column are responsible for collision warnings. The probabilities of the observed maneuvers using the CTRA motion model can be seen in Figure 4. The left side of the figure shows the probabilities regarding the lateral maneuvers. The right side is corresponding the longitudinals.

The accuracy of the model prediction is computed as follows. The most probable model for each motion model is considered the estimated maneuver at each timestep. These values, which are weighted by the mode probability, are compared to the predefined maneuvers—described in Table 1 and Table 2, resulting a prediction accuracy for each motion model. Table 7 describes the model accuracy of the diverse motion models comparing these. The first row denotes the classification precision of the different models regarding the longitudinal maneuver, while the second highlights laterals. The parameters are calculated as the ratio of time when the algorithm correctly classifies the maneuver.

**Figure 5:**
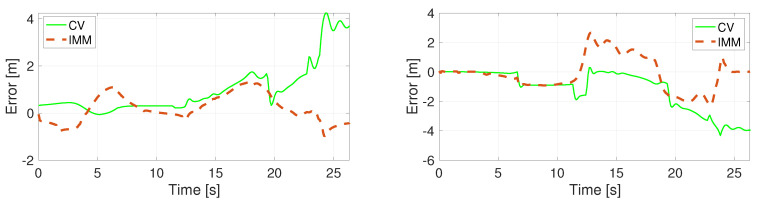
Position error in direction X (**left**) and Y (**right**) using the CV model.

**Figure 6:**
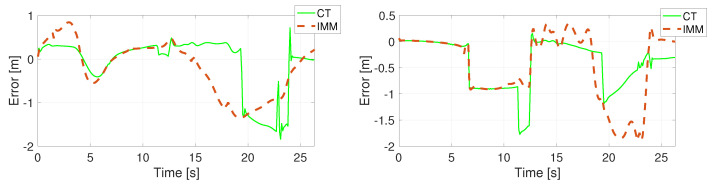
Position error in direction X (**left**) and Y (**right**) using the CTRV model.

**Figure 7:**
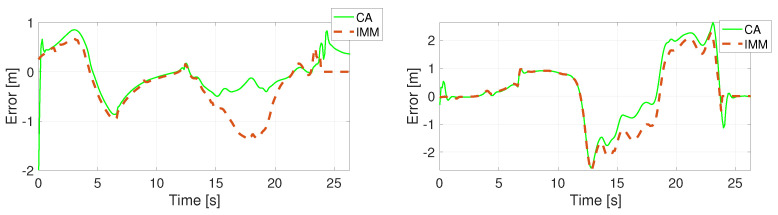
Position error in direction X (**left**) and Y (**right**) using the CA model.

**Figure 8:**
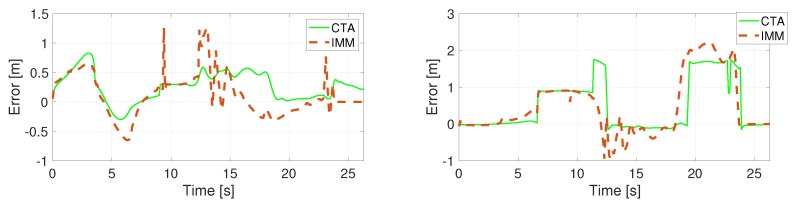
Position error in direction X (**left**) and Y (**right**) using the CTRA model.

The position error of the unconstrained filters using a specific motion model is calculated in the Euclidean space and presented in Figure 5, Figure 6, Figure 7 and Figure 8. Comparing the CV and the CTRV motion models, allowing yaw rate assures a better estimation, thus lower error.

The unconstrained estimators’ root mean square error (RMSE) is applied on both position components and presented in Table 8.

The two curvilinear motion models overcome the nonlinear models. CTRA and CTRV models present the best estimation with approximately 0.5 m error in the X and Y direction. In contrast, the estimation of the CV model comprises 1.5 m error. Even though the curvilinear motion models return with the best estimate, thus the lowest error, it is perceivable in Table 8 that the difference is negligible considering maneuver classification. As mentioned above in Section 3.2, constraints help reduce the uncertainty of a system, which is observable in this study likewise. Comparing CA and CTRA motion models, the above statement applies her too, although with lower contrast.

## 5. Conclusions

This paper presents a scenario-independent maneuver classification method, and its main purpose is to evaluate four different motion models in the given framework. The measurement and the state vectors must match, while the constraints must be defined separately. The presented algorithm utilizes the post-fit residual that profoundly enhances the accuracy of maneuver prediction. It is also combined with the IMM framework that enables the evaluation and comparison of the different motion models in a parallel manner. The algorithm uses constrained filters in the IMM structure and detects various maneuvers of an observed vehicle. The results of the maneuver classification are examined and evaluated based on four different motion models. It highlights the idea that from the aspect of the RMSE it is crucial to use the suitable motion model as the curvilinear models provide a significantly better position estimation than the linear. However, it is comprehensible that there is a modest difference between the models when constraints are applied as they alone reduce model uncertainty, as mentioned earlier.

A flexible solution is implemented; thus, the algorithm can be efficiently extended with new maneuvers. The IMM can face problems when the dimension of the various state spaces are not identical. In this study, a dimension extension method is introduced, where the state vector is extended to the same dimension with zero value. An other solution is mixing different dimension state vectors; thus, a more flexible system is implemented [36]. With nonlinear constraints more complex and refined maneuvers could be defined [33]. As the Kalman Filter has limited capabilities to handle nonlinearities, particle filter-based methods could be used instead to perform the filtering task [37]. The Variable Structure IMM (VSIMM) can handle variable model sets adaptively; thus, it can improve the system’s performance [38]. Baxter et al. [39] applied an adaptive motion model to track a person based on head-pose. Likewise, switching the motion model adaptively considering the performing maneuver can increase the system’s performance. 

## Figures and Tables

**Table 1:** Lateral maneuvers of the observed vehicle.

Maneuver	Start Time [s]	End Time [s]
Left lane	0	5
Lane change	5.1	6.4
Right lane	6.5	11.7
Lane change	11.8	12.6
Left lane	12.7	18.4
Lane change	18.5	19
Right lane	19.1	23.2
Lane change	23.3	23.9
Left lane	24	26.6

**Table 2:** Longitudinal maneuvers of the observed vehicle.

Speed	Start Time [s]	End Time [s]
Gaining distance	0	1.5
Collision warning	1.6	3.1
Distance keeping	3.2	4.1
Losing distance	4.2	8.5
Distance keeping	8.6	24
Losing distance	24	26.6

**Table 3:** Constraints for lateral maneuvers.

Mode	Position Constraint	Velocity Constraint
Right lane	y<l−0.5	$\dot{y}\;\epsilon \;N\left(0,1\right)$
Left lane	y>l+0.5	$\dot{y}\;\epsilon \;N\left(0,1\right)$
Lane change	l−0.5<y<l+0.5	$\dot{y}\;\epsilon \;N\left(0,1\right)$

where *l* denotes the lane width. These maneuvers define even the observed vehicle moves in lane or performing lane change. The constraint corresponding to the right lane consists of an upper limit, calculated by the width of the lane line. The left lane has lower limits, and the lane change maneuver constraint consists of upper and lower limits. The position constraints are introduced using hard inequality constraints and estimate projection. In contrast, the velocity component is derived as a zero-mean Gaussian because applying limitation is not required.

**Table 4:** Longitudinal maneuvers.

Maneuver	Velocity Constraint	Distance Constraint
Losing distance	$\dot{x}$ <= −1	*x* > 10
Gaining distance	$\dot{x}$ >= 1	*x* $\epsilon \;R^{+}$
Distance keeping	$\dot{x}$ = 0	*x* $\epsilon \;R^{+}$
Collision warning	$\dot{x}$ <= −1	*x* <= 10

**Table 5:** Constraint type of lateral maneuvers.

Maneuver	Type of Constraint	Estimation Method
Right lane	Hard inequality	Estimate projection
	Soft equality	Measurement augmentation
Left lane	Hard inequality	Estimate projection
	Soft equality	Measurement augmentation
Lane change	Hard inequality	Estimate projection
	Soft equality	Measurement augmentation

**Table 6:** Constraint type of longitudinal maneuvers.

Maneuver	Type of Constraint	Estimation Method
Losing distance	Hard inequality	Estimate projection
	Soft equality	Measurement augmentation
Gaining distance	Hard inequality	Estimate projection
	Soft equality	Measurement augmentation
Distance keeping	Soft equality	Measurement augmentation
Collision warning	Hard inequality	Estimate projection

**Table 7:** Accuracy of the models.

	CV	CTRV	CA	CTRA
lon	93.09%	91.73%	94.06%	93.91%
lat	89.59%	89.58%	89.74%	91.28%

**Table 8:** RMSE of the models.

	CV	CTRV	CA	CTRA
X	1.4684	0.6427	0.4297	0.3626
Y	1.8002	0.6139	1.1480	0.8427

## Data Availability

Source and data are available at the authors.

## Abbreviations

The following abbreviations are used in this manuscript:

CV	Constant Velocity
CA	Constant Acceleration
KF	Kalman Filter
CTRV	Constant Turn Rate and Velocity
CTRA	Constant Turn Rate and Acceleration
EKF	Extended Kalman Filter
MM	Multiple Model
IMM	Interacting Multiple Model
PF	Particle Filter
PDF	Probability Density Function
PM	Perfect Measurement
RMSE	Root Mean Square Error
VSIMM	Variable Structure Interacting Multiple Model
